# NGS-Based *S. aureus* Typing and Outbreak Analysis in Clinical Microbiology Laboratories: Lessons Learned From a Swiss-Wide Proficiency Test

**DOI:** 10.3389/fmicb.2020.591093

**Published:** 2020-11-24

**Authors:** David Dylus, Trestan Pillonel, Onya Opota, Daniel Wüthrich, Helena M. B. Seth-Smith, Adrian Egli, Stefano Leo, Vladimir Lazarevic, Jacques Schrenzel, Sacha Laurent, Claire Bertelli, Dominique S. Blanc, Stefan Neuenschwander, Alban Ramette, Laurent Falquet, Frank Imkamp, Peter M. Keller, Andre Kahles, Simone Oberhaensli, Valérie Barbié, Christophe Dessimoz, Gilbert Greub, Aitana Lebrand

**Affiliations:** ^1^Department of Computational Biology, University of Lausanne, Lausanne, Switzerland; ^2^Center for Integrative Genomics, University of Lausanne, Lausanne, Switzerland; ^3^SIB Swiss Institute of Bioinformatics, Lausanne, Switzerland; ^4^Institute of Microbiology, Lausanne University Hospital, University of Lausanne, Lausanne, Switzerland; ^5^Division of Clinical Bacteriology and Mycology, University Hospital of Basel, Basel, Switzerland; ^6^Applied Microbiology Research, Department of Biomedicine, University of Basel, Basel, Switzerland; ^7^Bacteriology Laboratory, Division of Laboratory Medicine, Department of Genetics Laboratory Medicine and Pathology, Geneva University Hospitals, Geneva, Switzerland; ^8^Service of Hospital Preventive Medicine, Lausanne University Hospital, University of Lausanne, Lausanne, Switzerland; ^9^Institute for Infectious Diseases, University of Bern, Bern, Switzerland; ^10^Department of Biology, University of Fribourg, Fribourg, Switzerland; ^11^Institute of Medical Microbiology, University of Zurich, Zurich, Switzerland; ^12^Biomedical Informatics, Swiss Federal Institute of Technology (ETH Zürich), ETH Zürich, Zurich, Switzerland; ^13^Interfaculty Bioinformatics Unit, University of Bern, Bern, Switzerland; ^14^Department of Genetics, Evolution and Environment, University College London, London, United Kingdom; ^15^Department of Computer Science, University College London, London, United Kingdom

**Keywords:** next generation sequencing, NGS, bacterial typing, SNP, ring trial, external quality assessment, EQA, quality control

## Abstract

Whole genome sequencing (WGS) enables high resolution typing of bacteria up to the single nucleotide polymorphism (SNP) level. WGS is used in clinical microbiology laboratories for infection control, molecular surveillance and outbreak analyses. Given the large palette of WGS reagents and bioinformatics tools, the Swiss clinical bacteriology community decided to conduct a ring trial (RT) to foster harmonization of NGS-based bacterial typing. The RT aimed at assessing methicillin-susceptible *Staphylococcus aureus* strain relatedness from WGS and epidemiological data. The RT was designed to disentangle the variability arising from differences in sample preparation, SNP calling and phylogenetic methods. Nine laboratories participated. The resulting phylogenetic tree and cluster identification were highly reproducible across the laboratories. Cluster interpretation was, however, more laboratory dependent, suggesting that an increased sharing of expertise across laboratories would contribute to further harmonization of practices. More detailed bioinformatic analyses unveiled that while similar clusters were found across laboratories, these were actually based on different sets of SNPs, differentially retained after sample preparation and SNP calling procedures. Despite this, the observed number of SNP differences between pairs of strains, an important criterion to determine strain relatedness given epidemiological information, was similar across pipelines for closely related strains when restricting SNP calls to a common core genome defined by *S. aureus* cgMLST schema. The lessons learned from this pilot study will serve the implementation of larger-scale RT, as a mean to have regular external quality assessments for laboratories performing WGS analyses in a clinical setting.

## Introduction

Hospitals and clinical laboratories are increasingly using next generation sequencing (NGS) technology to address a multitude of questions. Especially in clinical microbiology, whole genome sequencing (WGS) has been used for typing (cgMLST, SNP calling) [e.g., (Mellmann et al., 2017; Abdelbary et al., 2019; Zakham et al., 2019; Magalhães et al., 2020)], and enables addressing strain relatedness using high resolution data, e.g., for outbreaks within hospitals or in the community [e.g., (Deurenberg et al., 2017)], or at a larger geographic scale, e.g., for food-borne pathogens (Hendriksen et al., 2018), or other environmental pathogens [e.g., (Wüthrich et al., 2019)]. In addition, WGS data can provide very interesting information on the presence of specific resistance mutations, the acquisition of resistance genes (Ellington et al., 2017) or virulence factors (Tagini and Greub, 2017; Tagini et al., 2018).

NGS technology relies on complex laboratory workflows and generates high-throughput data that requires bioinformatic processing, analysis and interpretation. Proficiency tests (PT) have been implemented by ISO17043 organizations to address typing of *Staphylococcus aureus* in outbreak studies (e.g., qcmd.org). Current ISO-certified PT, however, do not focus on NGS-based analyses and cannot therefore be used as technical quality controls that would enable participants to benchmark their NGS workflows. In the meantime, several non-ISO-certified PT have been implemented. A multi-center ring trial comprising five laboratories to determine WGS-based typing of *S. aureus* showed very high reproducibility across laboratories for *spa* typing, MLST, rMLST, and cgMLST (Mellmann et al., 2017). The latter study did not address SNP calling and phylogenetic tree analysis. The Global Microbial Identifier (GMI) has been running several PT of which two editions, in 2015 and 2017, included *S. aureus* strains (Pedersen, 2017). The PT of 2015, for which a report is available (Pedersen, 2017), included analysis of SNP calling and cluster identification. More recently, in the Netherlands, a multicenter PT of bacterial outbreak analyses was implemented with 10 isolates each of methicillin-susceptible *S. aureus* strains, *Enterococcus faecium*, and *Klebsiella pneumoniae*, for which participants were asked to identify outbreak clusters from FASTQ datasets (personal communication from Jordy Coolen).

The Swiss Institute of Bioinformatics (SIB) leads and coordinates the field of bioinformatics in Switzerland, where it launched a nation-wide working group (WG) on NGS Microbes Typing and Characterization in 2016. The main aim of this WG was to harmonize NGS practices within Swiss clinical microbiology laboratories, especially with regards to bioinformatics. The WG includes microbiology and bioinformatics experts from all Swiss university hospitals and their associated clinical microbiology labs, cantonal hospitals, the Swiss Federal Institute for NBC-Protection (Spiez Laboratory), as well as research groups from Swiss academic institutions. It has met eight times face-to-face since its kick-off in September 2016, also running a RT on viral metagenomics (Junier et al., 2019).

A detailed survey on NGS practices at Swiss clinical microbiology laboratories conducted in 2017 highlighted a large variety of methodologies and software used to predict strain relatedness (not published). The WG therefore suggested that SIB (A. Lebrand) organizes in close collaboration with the Institute of Microbiology of the University of Lausanne (G. Greub) a Swiss-wide NGS-based bacterial typing ring trial, as a *technical* quality control test for assessing strain relatedness from WGS data. Such interest in participating to PT was also demonstrated worldwide by a Global Microbial Identifier (GMI) survey (Moran-Gilad et al., 2015). Building upon these other existing PT, the main objectives of the Swiss ring trial were to benchmark current workflows for *S. aureus* outbreak studies, by analyzing intermediary outputs (assemblies, typing, resistance, SNP calls, topology of trees, cluster identification, and cluster interpretation based on associated epidemiological data). With this design, we aimed to assess the impact of laboratory vs. bioinformatics variability on the intermediate outputs, to identify where knowledge/expertise sharing and training might be needed, and to define common best practices, with the ultimate goal to pave the way toward quality-controlled routine implementation of NGS-based bacterial typing in clinical microbiology laboratories through participation in external quality assessment (EQA) programs. We present here the results from the NGS bacterial typing ring trial that was run in Switzerland from November 2017 to July 2018.

## Materials and Methods

### Ring Trial Design

**FIGURE 1:**
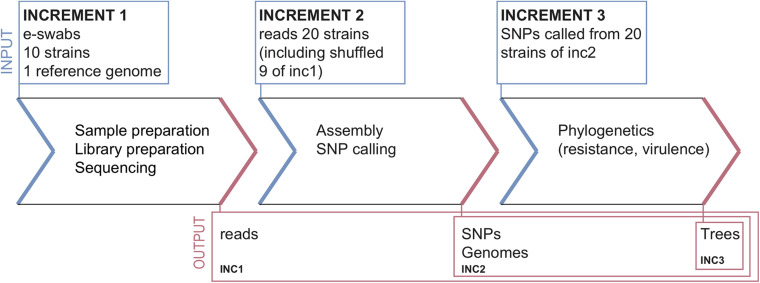
Ring-trial design.

The ring trial was designed to be a quality control test for assessing *S. aureus* strain relatedness from NGS data. It consisted of three parts, called increments (inc), selected to cover various parts of the NGS pipeline from sample preparation to reporting (Figure 1). The design aimed at disentangling the variability in the final outcome that might arise from differences in sample preparation, raw data processing for SNP calling and choice of phylogenetic methods.

#### Increment 1

Participants received:

• A questionnaire about the methodologies used in each pipeline (cf. section “Questionnaire” below).
• Ten bacterial strains and associated minimal epidemiological data (cf. section “Samples and Data for Increment 1”).
• A common *S. aureus* reference genome (cf. section “Common Reference Genome”).

Participants returned:

• Raw reads (FASTQ files), without any pre-processing, for every strain.
• Assembled genomes (FASTA files) for every strain.
• Unfiltered and filtered lists of identified SNPs for every strain (CSV or VCF files).
• Predicted resistance genes for every strain (optional).
• Phylogenetic tree including all the strains.
• Report interpreting the results (related strains, outbreak suspected or not).

#### Increment 2

Participants received:

• 20 FASTQ datasets consisting of ten samples from increment 1 (sequenced by the participants of increment 1, and re-labeled with a random number), and ten additional samples sequenced within the same sequencing facility (cf. section “Datasets for Increment 2”). Associated epidemiological data were also provided.

Participants returned the same data as for increment 1, except for the FASTQ files.

#### Increment 3

Participants received:

• Contigs (FASTA) and SNP calls (VCF) for the 20 strains from increment 2, labeled with the same numbers as in increment 2 (cf. section “Datasets for Increment 3”).

Participants only returned the predicted resistance genes (optional), a phylogenetic tree and a report.

### Ring Trial Implementation

The ring trial started in November 2017, and participants had 3 months to perform increment 1 (November 2017 to February 2018), 2.5 months for increment 2 (March to May 2018), and 2 months for increment 3 (June to July 2018).

Participants had the possibility to submit results obtained from several pipelines, where a pipeline is a specific combination of laboratory protocols and bioinformatic tools.

Six laboratories (represented by five sequencing centers) participated in increment 1, resulting in nine pipelines in total; in increment 2, three additional participants joined, for a total of nine laboratories and 12 pipelines; in increment 3, all laboratories from increment 1 participated (except one, which was not able to import VCF files into its tool). The methodologies used in all pipelines are briefly described in Table 1.

**TABLE 1:** Summary of pipeline methodologies. Each pipeline is represented by a capital letter. Note that several pipelines are based on the same NGS data, as only 5 sequencing facilities were used. NA, not applicable.

Sequencing facility	NA	I	II	II	II	NA	III	III	NA	IV	I	V

Pipeline	A	B	C	D	E	F	G	H	M	R	S	T
Bacterial growth	NA	NA	Columbia agar	Columbia agar	Columbia agar	NA	Columbia agar	Columbia agar	NA	Columbia agar	NA	MH agar
DNA extraction	NA	Wizard Genomic DNA Purification Kit Promega	EZ1/DNA tissue extraction kit	EZ1/DNA tissue extraction kit	EZ1/DNA tissue extraction kit	NA	UltraCleanÂ Microbial DNA Isolation Kit, MO BIO	UltraCleanÂ Microbial DNA Isolation Kit, MO BIO	NA	PureLink Microbiome DNA Purification Kit	Wizard Genomic DNA Purification Kit Promega	QIAGEN DNeasy Kit
Library preparation	NA	Nextera XT	Nextera XT	Nextera XT	Nextera XT	NA	QIASeq FX kit	QIASeq FX kit	NA	Nextera XT	Nextera XT	Nextera XT
Read length	NA	2 × 150 bp	2 × 300 bp	2 × 300 bp	2 × 300 bp	NA	2 × 150 bp	2 × 150 bp	NA	2 × 150 bp	2 × 150 bp	2 × 300 bp
Sequencing	NA	Illumina MiSeq	Illumina MiSeq	Illumina MiSeq	Illumina MiSeq	NA	Illumina MiSeq	Illumina MiSeq	NA	Illumina MiSeq + MinION	Illumina MiSeq	Illumina MiSeq
Assembly	SPAdes	NA	Velvet	Velvet	SPAdes	SPAdes	SPAdes	SPAdes	NA	Canu	SPAdes	SPAdes
SNP method/tool	BWA + bcftools	Bowtie	NA	CLC Genomics	NA	BWA + bcftools	BWA + VARSCAN	NA	BWA + snippy	Freebayes	Freebayes	BWA + Samtools
MLST tool	NA	Bionumeric	Ridom SeqSphere	CLC Genomics	NA	NA	ARIBA	NA	mlst 2.10	mlst 1.8	mlst 2.9	Ridom SeqSphere
Tree source	wgSNP	wgSNP	cgMLST	wgSNP	Core genes	cgSNP	cgSNP	cgMLST	cgSNP	wgSNP	cgSNP	wgSNP + cgSNP
Tree tool	FastTree	Bionumeric	Ridom SeqSphere	CLC Genomics	FastTree	SNPhylo	SeaView	Ridom SeqSphere	RaxML	MEGA7	RaxML	MEGA7
Tree Method	Neighbor-joining	UPGMA	Neighbor-joining	NA	Maximum-likelihood	Maximum-likelihood	Neighbor-joining	Neighbor-joining	Maximum-likelihood	NA	Maximum-likelihood	Neighbor-joining
Distance metric	GTR	Nb SNP differences	Allelic differences	Nb SNP differences	GTR	MUSCLE alignment	GTR	Allelic differences	Nb SNP differences	ANIm	GTR	Kimura 2-parameter
Resistance	Prokka (ResFam)	NA	Alere Microarray Resistance	NA	ABRicate	NA	ARG-ANNOT, RGI CARD, ResFinder	NA	Mykrobe	RGI CARD	RGI CARD, Mykrobe	Alere Microarray Resistance
												

Each pipeline was assigned a capital letter, whereas samples were numbered. Participants were asked to prefix output file names with the convention [pipeline_letter] + [sample_number], e.g., B5.fasta for the FASTA assembly of sample 5 resulting from pipeline B.

Participants also answered a short questionnaire on the methodologies used for each of the submitted pipelines (see section “Questionnaire” below). The questionnaire was protected by a pipeline-specific password, and let participants directly upload their small files to sync.com (password-protected) at the end of the questionnaire. For the larger FASTQ files, participants received a SWITCHfilesender voucher (50 Gb), SWITCH being the protected cloud of the Swiss academic community.

### Common Reference Genome

Participants received a common *S. aureus* reference genome in FASTA format (NCBI accession number NC_007795.1) to be used for read mapping and facilitate the comparison of SNP calls across pipelines.

### Samples and Data for Increment 1

Ten *S. aureus* strains were selected for increment 1 of the ring trial (Figure 1). These consisted of eight strains of Panton-Valentine leukocidin-producing *S. aureus* strains in asylum seekers (Jaton et al., 2016); and two strains from another, unpublished study. Bacteria were suspended in transport medium (E-swabs, COPAN Diagnostics, Ca), labeled with a number from 1 to 10, and shipped by mail with the instruction to grow the strains on solid medium to obtain colonies prior to analysis, according to each laboratory’s standard operating procedures. Participants were also encouraged to freeze and store the ring trial bacterial strains, for re-use as internal quality control, and also to enable further investigations during the ring trial if needed (e.g., suspicion of mislabeling or contamination). Samples for increment 1 were prepared by the Institute of Microbiology of the Lausanne University Hospital (CHUV-IMUL). Associated pseudonymized epidemiological data were also sent to participants, notably including date and site of isolation.

### Datasets for Increment 2

Twenty raw FASTQ datasets were provided to participants in increment 2 (Figure 1). Among those, ten corresponding to the strains provided in increment 1 were selected from among the sequencing data produced by the five sequencing centers of increment 1. Note that, while we intended to select FASTQ files representing the ten samples from increment 1, we realized afterward that one of the providing laboratories had swapped samples 2 and 3 in increment 1. Therefore, sample 3 was actually not present in increment 2, and sample 2 was provided twice, but sequenced by two different laboratories. There were therefore nine strains in common across all increments. Participants were not told from which laboratory the FASTQ files originated from, as the FASTQ headers were anonymized and cases were relabeled with a different number than in increment 1. Table 2 shows the correspondence of labels across all three increments.

**TABLE 2:** Sample labels in increments 1, 2, and 3; and origin of FASTQ datasets (generated in increment 1) used for increment 2.

Sample label		FASTQ origin
	
Inc. 1	Inc. 2 and 3	Inc. 2
1	8	S
2	14, 1	S, G
3	NA	*s*o*mp*/*e sw*o*p*
4	10	G
5	3	C
6	16	C
7	13	T
8	6	T
9	20	R
10	12	R
NA	2	CHUV-IMUL
NA	4	CHUV-IMUL
NA	5	CHUV-IMUL
NA	7	CHUV-IMUL
NA	9	CHUV-IMUL
NA	11	CHUV-IMUL
NA	15	CHUV-IMUL
NA	17	CHUV-IMUL
NA	18	CHUV-IMUL
NA	19	CHUV-IMUL

*Inc, increment; CHUV-IMUL, Institute of Microbiology of the Lausanne University Hospital; NA, not applicable.*

The remaining ten raw FASTQ datasets were provided by CHUV-IMUL. They were obtained from sequencing bacterial *S. aureus* strains on an Illumina MiSeq platform with paired-end 2 × 150 bp read length. Of these extra 10 cases, nine were methicillin-susceptible, and one was methicillin-resistant (sample 17 in increments 2–3).

Like for increment 1, associated pseudonymized epidemiological data were also sent to participants as a basis for cluster interpretation.

Datasets provided in increment 2, including pseudonymized epidemiological data, are available as Supplementary Material 1.

### Datasets for Increment 3

In increment 3 (Figure 1), we provided participants with assembled genomes (FASTA) and SNP calls (VCF) for the 20 strains from increment 2. Genomes were assembled using SPAdes 3.11.1 with standard parameters (Bankevich et al., 2012). SNPs were called using Snippy 3.2 (Seemann, 2015) mapped onto the common ring trial reference genome, similar to the approach taken in Jaton et al. (2016).

Datasets provided in increment 3, including pseudonymized epidemiological data, are available as Supplementary Material 2.

### Questionnaire

The questionnaire consisted of 24 questions covering:

• Storage
• Sample preparation
• DNA extraction, quantification, and quality assessment
• Library preparation
• Sequencing
• Bioinformatics (reads pre-processing, assembly, SNP calling, phylogenetics, resistance, and virulence)

The list of questions is available as Supplementary Material 3.

### Results Analysis

#### Sample Swapping

Pipeline G swapped samples 2 and 3 during increment 1. Thus, in the results from increment 1 presented here, we re-labeled samples correctly for pipeline G (i.e., swapped 2 and 3).

#### Contamination Analysis

We used Kraken (v0.10.6) (Wood and Salzberg, 2014) for the contamination analysis, using a database of 414 *S. aureus* strains and plasmids. To assess bacterial DNA contamination (by bacteria other than *S. aureus*), we assessed the percentage of reads that would *not* classify against this *S. aureus* strain database. To assess contamination by human DNA, we assessed the percentage of reads classifying against the human genome.

#### Assembly Analysis

Basic statistics were extracted using the assembly-stats tool (v1.0.1) (Sanger-pathogens, 2018) and the QUAST tool (v5.0.1) (Gurevich et al., 2013). We notably computed N50, L50, and depth of coverage as:

Number of reads × Average read length/genome length

where we used 2.8 Mbp as the reference genome size.

For gene prediction from assemblies we used prodigal v2.6.3 (Hyatt et al., 2010).

#### SNP Calling Analysis

Single nucleotide polymorphisms provided in VCF files were merged using bcftools (v.1.9) (Li et al., 2019). Since all submissions had slightly different formats, for each VCF file we parsed all the merged files and computed comma-separated files. In cases where for each strain a single file was given we used bgzip, bctools tabix and bcftools merge to generate a single file that contained all the positions. Participants could submit both unfiltered and filtered SNPs.

For downstream analyses, we discarded all non-SNP variants. Also, when both filtered and unfiltered SNPs had been provided by a pipeline, SNP comparisons were based on filtered SNPs. In addition, in order to facilitate comparisons between pipelines and strains, only SNPs located in the *S. aureus* core genome [defined here as the 1,861 locus part of *S. aureus* cgMLST (Leopold et al., 2014)] were considered when counting numbers of SNP differences between pairs of strains. For a given pipeline, when a SNP was called in one strain but was *not* in the other strain, we assumed a SNP difference between the two strains. Also, missing positions were assigned as reference. Note that we may, however, be overestimating SNP counts as some SNPs may be absent due to low sequencing depth or poor mapping quality in that particular region.

For a given strain, the similarity in SNP calls across pipelines was calculated using the Jaccard index, defined as:

$$J\,a\,c\,c\,a\,r\,d_{i\,n\,d\,e\,x}=\frac{\left|A\,\cap B\right|}{\left|A\,\cup B\right|}$$

where A represents the set of SNPs called by the first pipeline, and B the set of SNPs called by the second pipeline in the same strain.

#### Tree Analysis

Robinson–Foulds distance was computed using the ete3 (v3.1.1) library from python (v3.7). The normalized Euclidean distance was calculated using the python library DendroPy v4.4.0, by first normalizing each branch by the maximum distance between root to leaf and then calculating the Euclidean distance that is equivalent to the definition of branch length distance (Felsenstein, 2004). Distances were computed for each increment separately using all strains, and also for all increments combined using the nine strains common to all increments. Submissions that did not contain all provided strains were not considered for further analysis. For comparison of all increments, we trimmed the trees of increments 1, 2, and 3 of the strains that were not part of the nine common strains. For cluster comparison, we trimmed the trees for all strains not part of a cluster and collected branch lengths and pairwise distances.

#### Resistance Analysis

For each submission, we obtained a matrix in which the presence/absence of resistance genes for each strain was indicated. We then calculated the pairwise Pearson correlation between pipeline vectors as a measure of similarity between two pipelines and used this to obtain a hierarchical clustering and 2-dimensional spatial embedding using a principal component analysis.

All the analysis scripts and data are available for download as Supplementary Material 4.

## Results

The ring trial was designed to be a *technical* quality control test for assessing *S. aureus* strain relatedness from NGS data. It consisted of three increments (inc) selected to cover various parts of the NGS pipeline from sample preparation to reporting (Figure 1). The design aimed at disentangling the variability in the final outcome that might arise from differences in sample preparation, raw data processing for SNP calling and choice of phylogenetic methods (see section “Materials and Methods”).

**FIGURE 2:**
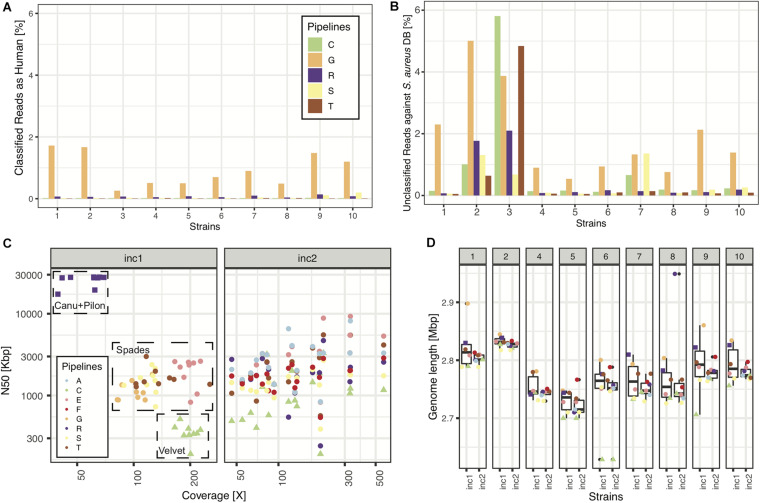
Sequencing and assembly quality (increment 1). In panels **(A,B)**, we show NGS quality analyses for the 5 pipelines representing the 5 sequencing facilities used in this RT. **(A)** Percentage of reads classified as having human origin, as a means to detect human contamination. **(B)** Percentage of reads unclassified against a database of >400 *S. aureus* strains. **(C)** N50 as a function of depth of coverage for all samples and pipelines. Note: pipelines **(C,D)** used the same assembly, thus only **(C)** is plotted, explaining why there are only 8 pipelines represented in panels **(C,D)**. **(D)** Assembly length distributions (across pipelines) for every strain of increment 1.

In increment 1, we obtained reads from five sequencing centers, all based on Illumina sequencing technology, except one that included a mix of MinION and Illumina reads (pipeline R). All read submissions had excellent quality values [mean(phred33 score) >33, data not shown] and low contamination levels that led to good assemblies (Figure 2; see section “Results” in Supplementary Material for more details). As reported in another ring trial (Mellmann et al., 2017), we also found perfect agreement between clinical laboratories whenever a sequence type was called (Table 3; see section “Results” in Supplementary Material for more details).

**TABLE 3:** Identified MLST for the 10 strains of increment 1.

Pipeline, tool		1	2	3	4	5	6	7	8	9	10
C	Seqsphere+ 4.1.9	ST5	U	ST45	ST15	ST152	ST152	ST15	ST15	U	U
D	CLC Genomics 10.1.1	ST5	ST121	ST45	ST15	ST152	ST152	ST15	ST15	U	U
G	ARIBA 2.10.2	ST5	ST121	ST45	ST15	ST152	ST152	ST15	ST15	U	U
R	mlst server 1.8	ST5	ST121	ST45	U	U	ST152	ST15	U	U	U
S	mlst server 2.9	ST5	U	ST45	ST15	ST152	ST152	ST15	ST15	U	U
B	Bionumerics 7.6.3894	ST5	U	ST45	ST15	ST152	ST152	ST15	ST15	U	U
T	Seqsphere+ 4.1.9	ST5	U	ST45	ST15	ST152	ST152	ST15	ST15	U	U

*Missing pipelines did not submit any MLST. U, unknown; note: samples 9, 10 correspond to ST5685, unknown at the time of the study.*

### The Observed Number of SNPs Differences Is Robust to Experimental Variability for Closely Related Strains

Due to their high resolution compared to MLST, SNP calls obtained from NGS data can be used to assess strain relatedness in a suspected outbreak, when combined with additional epidemiological information. Indeed, the expected number of SNP differences between any two related strains is expected to increase with time, as the strains will evolve at some mutation rate. In *S. aureus*, the mutation rate has been estimated to be around one core SNP per 6 weeks (Harris et al., 2010).

Given the importance of SNP calling in the process of assessing strain relatedness, we investigated the robustness of SNP calls across pipelines, and of the observed number of SNP differences between any pair of strains across pipelines. In order to facilitate comparisons, we asked participants to call SNPs against a common reference genome that we provided.

**FIGURE 3:**
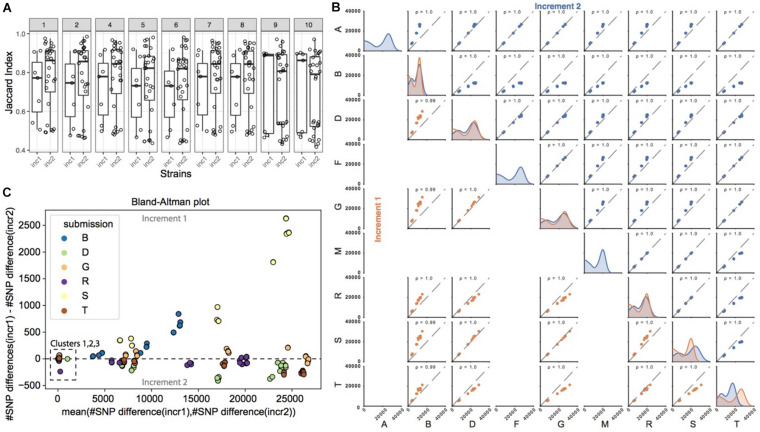
SNP calling pipelines show significant variation in the identified SNP positions. **(A)** Pairwise pipeline comparison. For each strain and increment, we plotted the percent overlap in SNP positions between any pair of pipelines (each dot represents a pair of pipelines; the percent overlap is defined as the Jaccard index). **(B)** Pairwise pipeline comparison of the number of SNPs differences for each pair of strains (note that only 9 pipelines reported SNPs; please refer to Table 1 “SNP method/tool”). For every pair of strains, we calculated the pairwise number of SNP differences. Two closely related strains are expected to have a small number of pairwise SNP differences. Plots in the diagonal of the matrix show the distribution of the number of SNP differences between all pairs of strains, for a given pipeline. All pipelines generally highlighted a bimodal distribution, with a first peak with smaller number of SNP differences for more closely related strains, and a second peak corresponding to more distantly related strains. Colors refer to the increment (orange for increment 1, blue for increment 2). Plots outside the diagonal of the matrix represent the number of SNP differences between pairs of strains, for two given pipelines (each dot represents the number of SNP differences between a pair of strains, in one pipeline versus the other pipeline). If two pipelines consistently identified very similar numbers of SNP differences between all strains pairs, then the data points will be perfectly correlated, as exemplified e.g., in increment 1 for pipelines D and S. **(C)** We show here the comparison of pairwise SNP differences across increments, for a given pipeline. Out of the 9 pipelines reporting SNPs (Table 1), only 6 submitted SNPs to both increments 1 and 2 and are represented here. For every pair of strains common to increments 1 and 2, we plot on the *y*-axis the number of SNP differences in increment 1, minus the number of SNP differences for that same pair of strains in increment 2. Deviations from zero denote that the SNP calling pipeline was sensitive to the experimental conditions (sample and library preparation, sequencing). Data points are represented against the average number of SNP differences in increment 1 and increment 2 for the corresponding pair of strains (*x*-axis).

Figure 3A shows the similarity in SNP calls between all pairs of pipelines, for each of the strains common to increments 1 and 2. The similarity was calculated using the Jaccard index, which for a given strain takes the number of SNPs observed in common between a pair of pipelines, and divides it by the total number of SNPs called by this pair of pipelines (see section “Materials and Methods” on how SNP calls were made comparable across pipelines). It therefore reflects the ratio of SNPs in common between these two pipelines. Even though participants had the same strains and a common reference genome, we observed that the Jaccard index was rather low in increment 1, with a median just above 50%. In order to investigate the impact of sample preparation and sequencing on SNP calling, we then compared the Jaccard indices from increments 1 and 2. In general, for strains 1 to 8, we observed a shift in the distribution of the Jaccard indices, with higher overlap of SNP calls between several pairs of pipelines in increment 2 compared to increment 1 as shown by the much higher median above 80%, suggesting that differences in SNP calls across pipelines were in part due to different experimental procedures. We note, however, that the variability in Jaccard indices across pairs of pipelines was still very high as denoted by the still large interquartile range, suggesting that bioinformatics procedures also contribute to differences in SNP calls between pipelines.

Since it is generally the *number* of SNP differences between pairs of strains that is used along with epidemiological information to infer strain relatedness and potential transmission links, we wondered if pipelines, despite calling different sets of SNPs, would nevertheless predict similar number of pairwise SNP differences between any pair of strains, and in particular if they identified the same closest strains with roughly the same number of SNP differences between those strains (i.e., comparable order of magnitude). In Figure 3B (plots in the diagonal of the matrix), we observed that most pairs of strains exhibited several thousand SNP differences, and that only a few pairs of strains had smaller number of SNP differences (bimodal distribution), consistent with the fact that only few strains were more closely related. We then investigated if there was a correlation between the *number* of SNP differences between pairs of strains, as observed by each pipeline (Figure 3B, scatter plots). In brief, if two pipelines were to predict the same number of SNP differences for all the possible pairs of strains, then the data points (representing pairs of strains) would follow the diagonal. Data points outside the diagonal would highlight pairs of strains for which the two pipelines differed in their prediction of number of SNP differences, e.g., one pipeline predicting two closely related strains, and the other predicting instead more SNP differences between those two same strains.

In both increments, the Pearson correlation was always close to 1. Data points, however, tended to shift away from the diagonal (in particular when comparing pipeline B against the others), meaning that the predicted absolute number of SNP differences between pairs of strains differed between the pipelines, although ranking was preserved. In order to better understand those differences, we plotted the same information in the form of Bland-Altman plots [Supplementary Figure 1 (increment 1) and Supplementary Figure 2 (increment 2)]. Interestingly, we observed that only the more distantly related pairs of strains tended to deviate from zero, showing that pipelines generally agree with one another on the absolute number of SNP differences for very closely related strains harboring <100 SNPs differences, and less so for strains that are more distantly related.

In order to investigate the potential impact of sample preparation and sequencing on the number of SNP differences, we then focused on the nine strains common to increments 1 and 2. Except for pipeline S, we did not observe significant changes in the number of pairwise SNP differences for any given pipeline from increment 1 to increment 2 (Figure 3C), showing consistency and robustness in SNP calls for a given pipeline, despite experimental variability. This suggests that variability arising from experimental procedures did not alter the final number of SNP differences called by each individual pipeline between any two strains.

In summary, different pipelines called different sets of SNPs both due to experimental and bioinformatics procedures, but ultimately, the observed number of SNPs *differences* between any two strains was robust to experimental variability for the closely related strains.

### Clinical Laboratories Consistently Group the Right Strains in Clusters

**FIGURE 4:**
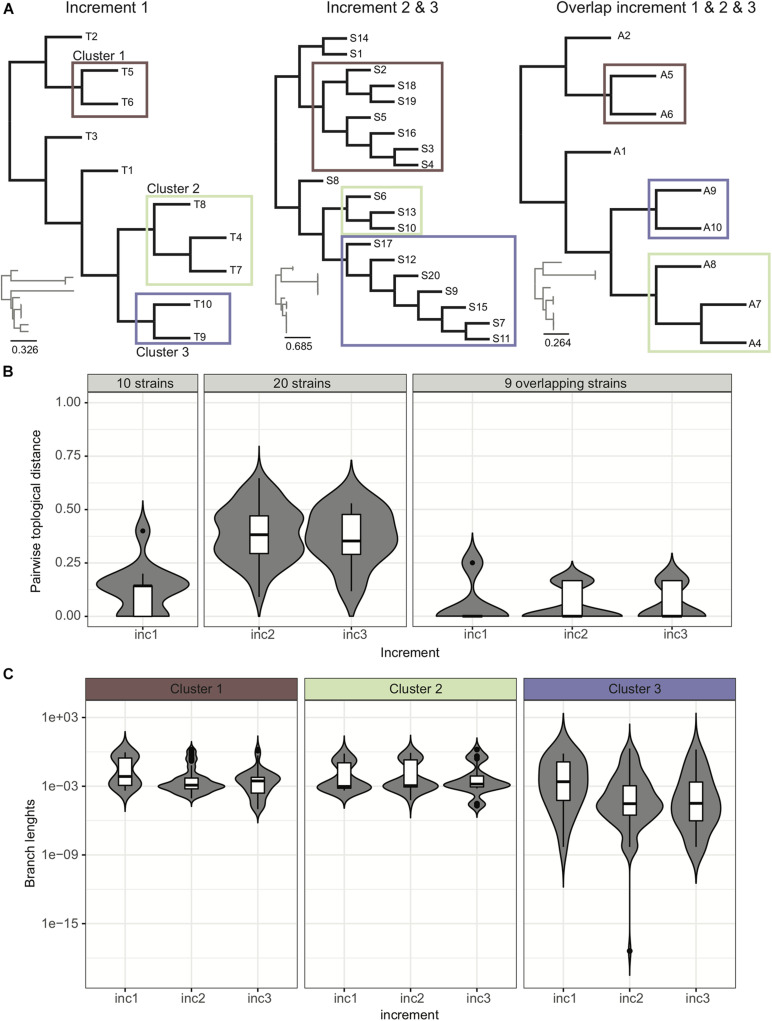
Analysis of trees. **(A)** Example of trees submitted by participants for the various increments. We were able to identify four distinct groups of trees (see Supplementary Figure 3). **(B)** Pairwise topological distance using all the data and the overlapping strains. We represent the density of pairwise topological distances in the form of boxplots, and also show in the background the violin plot of the actual density from which the boxplot was drawn, highlighting in some cases multi-modal distributions. Although when considering the 20 strains there is a high dispersion of pairwise topological distances, this dispersion actually occurs due to the topological changes *within* the clusters of strains (as reported by participants in the ring trial) (Supplementary Figure 4). For trimmed trees containing only the nine strains common to all increments, the dispersion of topological pairwise distances is reduced. **(C)** Variance of branch lengths for extracted branches from clusters. See also Supplementary Figure 5.

Phylogenetic trees provide an easy way to distinguish and visualize clusters of related strains that may be part of an outbreak. An outbreak in a tree would be characterized by the presence of a clearly distinguishable subtree with extremely short branches. Trees may also be decorated with additional information like patient proximity in the hospital or presence/absence of resistance/virulence factors, to facilitate data interpretation and outbreak surveillance. In this ring trial, participants were asked to submit phylogenetic trees in all three increments. Given the provided strains per increment, we expected the participants to identify three clusters of related strains, although not part of an outbreak, these being too distantly related and belonging to clusters of circulating strains, or a consequence of likely laboratory contamination (Figure 4A).

Our data indicates that cluster identification (as reported by participants) was robust. When further investigating topological variance across the trees submitted by the participants, we observed that variance in topologies was mainly due to variations *within* the subtrees (Figures 4B,C; see section “Results” in Supplementary Material for more details).

### Interpretation of Results Is More Expertise-Dependent

Participants were asked to submit a report interpreting their results at the end of each increment. We discuss here in more detail the nine reports submitted at increment 1 (6 laboratories, 9 pipelines), which reflect the expertise from laboratories associated with the five Swiss University Hospitals (clinical microbiology and infection control).

Participants reported their interpretation with free text in a document. We observed that wordings were not standardized and even sometimes confusing. For example, terms to qualify a cluster as likely not an outbreak encompassed “no direct transmission,” “unlikely common source,” “likely not an outbreak,” “very unlikely recent transmission or acquisition from same source,” “not compatible with a common source of strain transmission,” “level of variation superior to that expected in case of an outbreak.” One report also used the wording “have a common source,” which could be interpreted as reporting an outbreak, although the participant clarified later on that he/she did not mean to report an outbreak. Wordings to report a likely outbreak included “suspicion of direct transmission,” “may be clonal,” “could be an outbreak considering epidemiological info.”

Since participants always reported identical interpretations for all the pipelines for which they were submitting results, we present in Table 4 the interpretations as a function of the number of pipelines, but also as a function of the number of laboratories. Cluster 1 (strains 5, 6) was correctly reported as not an outbreak by 5 out of 6 laboratories, as was cluster 2 (strains 4, 7, 8) by 4 out of 5 laboratories (note that different laboratories suggested that clusters 1 and 2 may represent an outbreak). Lastly, as indicated in the epidemiological information provided to participants in increment 1, cluster 3 (strains 9, 10) likely resulted from a lab contamination. Interestingly, this was correctly spotted by seven pipelines (4 laboratories), but two pipelines (and laboratories) missed that epidemiological information and reported a potential direct transmission.

**TABLE 4:** Cluster interpretation.

	Number of pipelines			Number of laboratories		
		
	Cluster 1	Cluster 2	Cluster 3	Cluster 1	Cluster 2	Cluster 3
No direct transmissions	8	6	0	5	4	0
Suspicion of direct transmission	1	2	2	1	1	2
Likely lab contamination	0	0	7	0	0	4

*Note that pipeline R, based on MinION data, did not fully identify cluster 2, since strain 4 was missing from its tree. Hence, we only evaluated eight pipelines and five laboratories for cluster 2.*

It is interesting to note that reports were not always quantitative in their interpretation. Thus, while some clearly mentioned expected number of SNP differences given the dates of isolation (Harris et al., 2010), others justified their conclusion with qualitative terms (e.g., “few genomic differences”). In summary, while cluster identification was very robust across pipelines, their interpretation remained more expertise-dependent, highlighting the need for harmonization.

### Prediction of Acquired Resistance Genes

This ring trial was mainly designed as a quality control for assessing strain relatedness and outbreak detection. We however, took the opportunity to also ask participants to predict acquired resistance genes if they wished. We did not validate experimentally the presence of the reported genes, but two participating accredited clinical laboratories performed phenotypic antibiotic susceptibility testing (AST) on the ten strains from increment 1 and shared their results (Supplementary Table 2). The range of antibiotics tested was not identical in the two laboratories, but both of them found that all strains were penicillin resistant, and that strain 4 was in addition resistant to tetracycline. One of the two laboratories identified strain 7 as tetracycline-resistant.

Antimicrobial resistance (AMR) was predicted using various tools and databases (cf. Table 1) [Alere Microarray, CARD (Jia et al., 2017), Mykrobe (Bradley et al., 2015), ARG-ANNOT (Gupta et al., 2014), ResFinder (Zankari et al., 2012)]. Some pipelines actually returned results from various tools, resulting in various “resistance subpipelines”. Thus, we ended up with eight resistance subpipelines in increment 1, and nine in increment 2. There were 25 resistance genes detected by at least one of the resistance subpipelines. Nearly every subpipeline detected *blaZ* in all strains, as expected from phenotypic AST (Supplementary Figure 6). In addition, *tetK* was identified by seven of the eight subpipelines in both strains 4 and 7, suggesting that strain 7 might indeed be tetracycline resistant as phenotypically measured by one of the two laboratories performing AST. The trimethoprim resistance gene *dfrG* was identified by almost all subpipelines in strains 1, 4, 7, 8 but the resistance to this antibiotic was not phenotypically tested. The remaining 22 genes were identified by 1 to 4 subpipelines only. Several of these genes are likely to confer resistance to antibiotics that were not tested phenotypically in our study, or participate in general multidrug resistance mechanisms (e.g., efflux, inactivation, plasmid partition), precluding the direct link from genotypically predicted AMR to phenotypic AST. In summary, for those genes expected considering the results from the phenotypic ASTs, almost all the subpipelines agreed on their presence.

**FIGURE 5:**
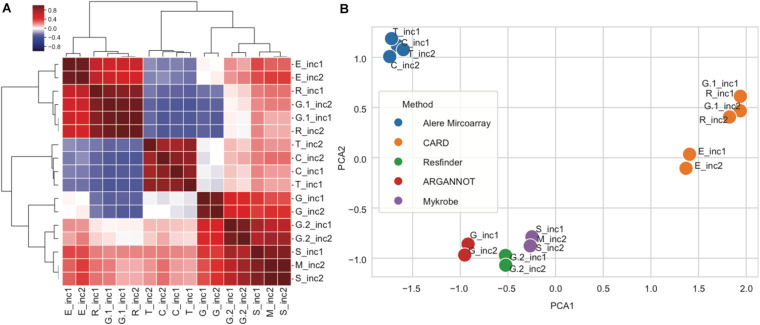
Correlations between subpipelines for antibiotic resistance gene detection. **(A)** Hierarchical clustering of pairwise correlations between subpipelines (dark red: positive correlation 1 and dark blue negative correlation −1). **(B)** Principal component analysis of pairwise correlation matrix overlayed with method used for resistance prediction.

In order to further explore the variability between subpipelines, we computed the correlation in predicted genes between any pair of subpipelines using the nine overlapping strains from increments 1 and 2. For this, every subpipeline was represented by a matrix, where every row is a gene and every column is a strain, and the value is 1 for presence and 0 for absence of the corresponding gene. To compute similarity, we then computed the correlation between any pair of subpipelines matrices, resulting in the similarity matrix shown in Figure 5A. We then grouped together rows and columns by hierarchical clustering (Figure 5A), and performed principal component analysis (PCA) (Figure 5B).

We observed that the various subpipelines consistently detected the same set of genes in strains throughout increments 1 and 2, as shown by the fact that “_inc1” and “_inc2” for a given subpipeline were always found close to each other on the PCA projection (Figure 5B). Moreover, we saw a clear separation of subpipelines depending on the database/method that was used (Figure 5B): Alere Microarray Resistance and CARD were distinctly separated from Mykrobe, ARG-ANNOT and ResFinder. Especially, CARD approach resulted in the highest number of predicted genes, as compared to all the other approaches (17/25).

## Discussion

The RT that we implemented within the Swiss NGS bacterial typing community was aimed at harmonizing NGS practices for bacterial typing and outbreak investigations in a clinical setting. By harmonizing, we mean for different clinical laboratories to achieve comparable results of high quality, independent of the methodology and tools chosen at the experimental and bioinformatics levels. Indeed, some experimental choices may, for example, result from internal constraints, such as using the existing laboratory nucleic acid extraction protocol. In order for others to also benefit from our study and the data that we generated, we provide the datasets and epidemiological information that we generated (Supplementary Materials 1,2). In Switzerland, this RT was very useful in that it enabled Swiss clinical microbiology laboratories to rapidly agree on the common bioinformatics analysis pipeline to be implemented for Illumina NGS data on the Swiss Pathogen Surveillance Platform (spsp.ch), a secure One-health online platform in testing phase that enables near real-time sharing under controlled access of pathogen whole genome sequences (WGS) and their associated clinical/epidemiological metadata (Egli et al., 2018).

Our results indicate that most Swiss clinical microbiology laboratories make use of Illumina sequencing technology, and that the sequencing is of very high quality, with low contamination levels (Figure 2). Laboratories using the same assembly tool (SPAdes being the most common) also achieved very similar assembly quality metrics with Illumina data. Since only one laboratory used long reads, no comparisons can be made for that technology.

Overall, we observed that MLST typing results, phylogenetic tree construction and cluster identification were highly robust across laboratories, despite the different experimental and bioinformatics workflows. Output results were also robust for pipelines with higher contamination levels (cf. Figures 2A,B). However, for outbreak investigation, our results suggest that differences in interpretation can yield to different conclusions. Importantly, different laboratories used different wordings and qualitative vs. quantitative criteria to state their clinical interpretations. From this ring trial, it appears that knowledge-sharing and definition of common, quantitative, interpretation criteria would be essential ingredients for harmonizing NGS practices, thereby enabling comparable, easily-understandable, interoperable and intra- and inter-laboratory reproducible conclusions.

To our surprise, the high level of agreement in tree construction and cluster identification, however, hid a rather poor overlap in the observed sets of SNPs used to build the tree (Figure 3A). Thus, while pipelines’ final output trees and clusters were highly intra-laboratory reproducible and robust to experimental procedures (Figure 4), the sets of identified SNPs used to determine strain relatedness actually differed from one pipeline to another (Figure 3A). This is explained by the fact that the *number* of SNP differences between strains correlated well across pipelines (Figure 3B and Supplementary Figures 1, 2), meaning that closer strains had fewer SNP differences in all pipelines, whereas more distantly related strains had a higher number of SNP differences in all pipelines. We note here that we only investigated pairwise SNP differences, but that pairs of strains may harbor other mutations such as insertions and deletions.

Our analysis therefore indicates that bioinformatics tools can have a great impact on SNP calls and, for more distantly related strains, on the number of pairwise SNP differences between strains. We would recommend restricting SNP calls to a common core genome (e.g., as defined by the cgMLST schema or common to the investigated strains) and filtering them as a means to more robustly exchange data on SNP calls and number of SNP differences between different laboratories using different bioinformatics tools.

Regarding antimicrobial resistance prediction, we observed that up to 25 genes were predicted using various *in silico* resistance prediction tools, with *blaZ* and *tetK* genes showing high concordance between tools and with phenotypic AST, whereas most of the other predicted genes showed little concordance across tools, largely due to differences in databases and the number of genes contained within. This notably calls for clinically curated databases of AMR, and better assessment of the tools to be used and how predictions should be combined to achieve highly accurate gene detection.

Switzerland is a small country with few laboratories performing NGS for outbreak analyses, explaining the small sample size in our pilot RT. The lessons learned in this RT will, however, be useful for the development of larger-scale international technical RT to serve as benchmarking and regular quality control tests for laboratories performing NGS analyses in a clinical setting. Participation in such quality controls are indeed mandatory for the use of NGS in accredited diagnostics laboratories.

## Data Availability Statement

The datasets presented in this study can be found in online repositories. The names of the repository/repositories and accession number(s) can be found in the article/Supplementary Material.

## Author Contributions

DD, TP, OO, GG, and AL: methodology. DD: formal analysis and visualization. TP, OO, and GG: resources. DW, HS-S, AE, SLe, VL, JS, SLa, CB, DB, SN, AR, LF, FI, PK, AK, and SO: investigation. DD and AL: writing – original draft. TP, OO, DW, HS-S, AE, SLe, VL, JS, SLa, CB, DB, SN, AR, LF, FI, PK, AK, SO, VB, CD, and GG: writing – review and editing. AL: supervision and project administration. All authors: conceptualization.

## Conflict of Interest

The authors declare that the research was conducted in the absence of any commercial or financial relationships that could be construed as a potential conflict of interest.
