# Supplementary material for: NGS-Based S. aureus Typing and Outbreak Analysis in Clinical Microbiology Laboratories: Lessons Learned From a Swiss-Wide Proficiency Test
Source: Front Microbiol. 2020 Nov 24;11:591093. doi: 10.3389/fmicb.2020.591093 (PMC7793906; doi:10.3389/fmicb.2020.591093)
Supplement: Supplementary file 1 [file Data_Sheet_1.pdf]

Supplementary Material

Results 1

Contamination and assembly quality 1

MLST typing 2

Tree topologies 3

Supplementary Figures 5

Supplementary Tables 10

Supplementary Materials 12

Results

Contamination and assembly quality

We investigated the level of laboratory contamination during sample preparation. In total, five sequencing facilities were used by participants (note that several laboratories submitted results obtained with various pipelines but based on the same FASTQ data, explaining why we have more pipelines than sequencing facilities; cf. Table 1). In Fig. 2A-B, we show NGS data quality results for 5 pipelines representing the 5 sequencing facilities. Except pipeline G showing higher levels of contamination from human DNA, all the other sequencing facilities showed levels of human contamination below 0.25% (**Fig. 2A**). We also observed that pipeline G had in general higher levels of reads *not* mapping against *S. aureus* genomes (**Fig. 2B**). Interestingly, strains 2 and 3 showed higher levels of unclassified reads for all pipelines, suggesting that this may not be due to laboratory contamination, but inherent to these strains (e.g. plasmid).

The average depth of coverage varied from ~40X to ~200X (**Fig. 2C**). When plotting depth of coverage against N50, we did not observe any significant improvement in N50 when increasing sequencing depth, for neither Canu+Pilon, SPAdes or Velvet based analyses. Pilon assemblies obtained from the MinION long reads showed the highest N50 values despite low coverage, consistent with the fact that a combination using long MinION and short Illumina reads generally performs better in this statistic. Additionally, we observed that Velvet assemblies showed lower N50 values, and this was also consistent in increment 2.

In increment 2, the same FASTQ datasets were provided to all participants. Interestingly, although most assemblies in increment 2 were generated using the tool SPAdes, we still saw variations in N50 across the pipelines of one to two orders of magnitude, highlighting the impact of parameters setting.

We also looked at assembly length as a means to assess assembly quality across pipelines (**Fig. 2D**). When comparing the assemblies for the nine strains common to increments 1 and 2, we observed a similar median in genome length across the different pipelines, although some pipelines, notably that based on MinION data, did report very different genome lengths. Altogether, our observations indicate that throughout Switzerland the sequencing facilities provided clean reads, that coverage beyond 50X does not lead to significant improvements in various statistics and that SPAdes assemblies in general performed best when using Illumina short reads.

MLST typing

Multi-locus sequence typing (MLST) has become a standard for bacterial typing based on a species-specific schema, and has been used as a tool for global epidemiology (10). In this RT, participants could submit the identified sequence type (ST) in the online questionnaire. We report in **Table 3** the results obtained by participants for the ten strains of increment 1. As reported in another ring trial (11), we found perfect agreement between clinical laboratories whenever a ST was called. Interestingly, we observed that some pipelines could predict the consensus ST for more strains (where the consensus is defined from the participants submissions). Considering all ten strains, pipelines D and G that used CLC Genomics and ARIBA, respectively, were able to call the consensus ST in 8 out of 10 strains. Note however that in increment 1, participants did not start from the same FASTQ data, and thus, we cannot exclude that differences between the various MLST tools may also be due to differences in read quality. To further test this, we looked at the MLST predictions in increment 2, where participants received the same 20 FASTQ datasets (**Supplementary Table 1**). We observed that pipeline R now predicted the consensus ST for strains for which it had reported no ST in increment 1. Considering that this laboratory reported having sequencing quality issues for those two strains on MinION, these results highlight the importance of sequencing quality for downstream analyses such as MLST calling. In increment 2, all the pipelines agreed on the ST of all isolates, except for strains (1,14)

corresponding to strain 2 of increment 1, where some pipelines reported an unknown ST, whereas others reported ST121 as the closest ST (6,5,6,2,7,14,5*), highlighting different reporting practices.

Tree topologies

Participants mostly generated trees based on SNP calls (core SNPs or whole genome SNPs), but also used alignment of core genes or core genome allelic differences represented in a minimum spanning tree (MST) as means to compute distances between strains (**Table 1, Fig. 4A**). We assessed whether the various tree topologies could be grouped by specific features of the various pipelines. We compared trees pairwise by considering variations in branch length in addition to topology, and used only the nine strains common across all three increments. We were able to identify four distinct groups of trees (**SFig. 3**). The two main groups could be divided by the choice of distance metric used to compute the tree, which was either (i) the number of allelic differences from cgMLST (pipelines C, H), or (ii) the number of core/whole genome SNPs differences, or GTR (Tavaré S 1986) distance between core genes or core/whole genome SNPs. The remaining two outgroups were formed by pipelines with a different phylogenetic methodology. Pipeline R in increment 1 used low coverage read data (MinION) and the average nucleotide identity (ANIm) of matching regions following NUCmer alignment as a distance metric, and was lacking strains 4 and 5 (inc. 1) in the tree because of poor quality sequencing. Pipeline F used MUSCLE to align the nucleotide sequences, which is based on a sum-of-pairs score using a scoring scheme taken from BLASTZ (Edgar 2005).

To assess topological variation across the pipelines, we computed the Robinson-Foulds distance between each pair of trees, which counts the number of different splits between two trees (**Fig. 4B**). For increment 1, most trees had a distance of zero, thus being identical. However, for increments 2 and 3, we observed mean topological pairwise distances larger than zero, indicative for topological variations across pipelines. In order to characterize the source of variation, we computed the topological distance of all trees of all increments trimmed to the nine overlapping strains. When considering the nine-strain trees, the mean topological variation approached zero (**Fig. 4B**). Therefore, the inference of the backbone of the subtrees was robust throughout the pipelines, suggesting that for increments 2 and 3, the source of variation of topological differences was due to changes within the subtrees. To address our hypothesis, we computed the topological distance

between a pair of subtrees excised from their respective full tree (defined in **Fig. 4A**). We indeed observed a high dispersion of pairwise topological distances with a high mean (**SFig. 4**). The lack of phylogenetic signal to reliably compute the right splits might be due to the close relatedness of the strains and the small amount of SNP differences between them (compare to **Fig. 3**). In summary, our data indicates that cluster identification (as reported by participants) was robust and that variance in topologies was mainly due to variations *within* the subtrees.

With the incremental design of our ring trial, we reduced the degrees of freedom throughout the increments, and thereby reduced the various sources of individual variation along the workflow. Therefore, we would expect results to become more similar throughout increments. Since branch lengths are used as an indicator of the evolutionary distance between two strains, we investigated the robustness of branch lengths to variability in the laboratory and bioinformatics workflows. Thus, we assessed whether the distributions of branch lengths across pipelines showed a decrease in variation along the increments. Specifically, we looked at the variation of branch lengths within the subtrees. When accumulating all possible branch lengths per subtree and per increment, we see a similar mean with comparable standard deviations across increments. Indeed, the distribution of branch lengths across increments for each subtree remains constant with little variation (**Fig. 4C**). This is, furthermore, true for most but not all pipelines (**SFig. 5**). In summary, while SNP calls were very much dependent on the bioinformatics tools, we observed that the identification of clusters (i.e. subtrees) was robust across pipelines and increments, and that branch lengths *within* clusters were also robust across increments.

Supplementary Figures

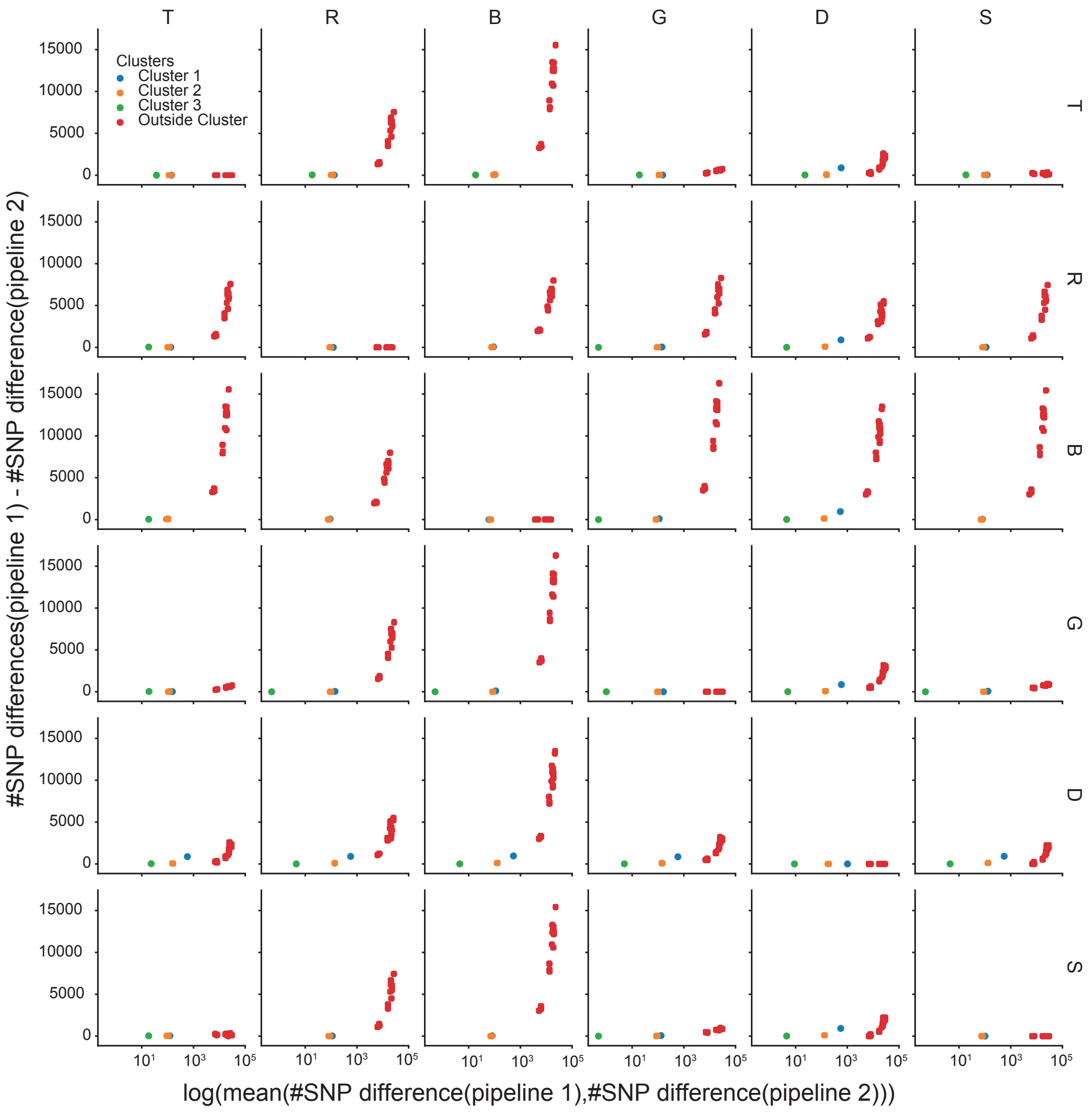

Supplementary Figure 1. *Comparison of pairwise SNP differences across pipelines (increment 1; note that only 6 pipelines from increment 1 reported SNPs (Table 1))*. Every subplot is the comparison between two pipelines, where we plot on the y-axis the absolute value of the number of SNP differences for a pair of strains in the first pipeline, minus the number of SNP differences for that same pair of strains for the second pipeline. Values close to zero indicate that the two pipelines agreed on a similar number of pairwise SNP differences for that pair of strains (see also Fig. 3B, which is essentially another representation of the same data). Data points are represented against the average of the number of SNP differences in the first and second pipeline for the corresponding pair of strains (x-axis).

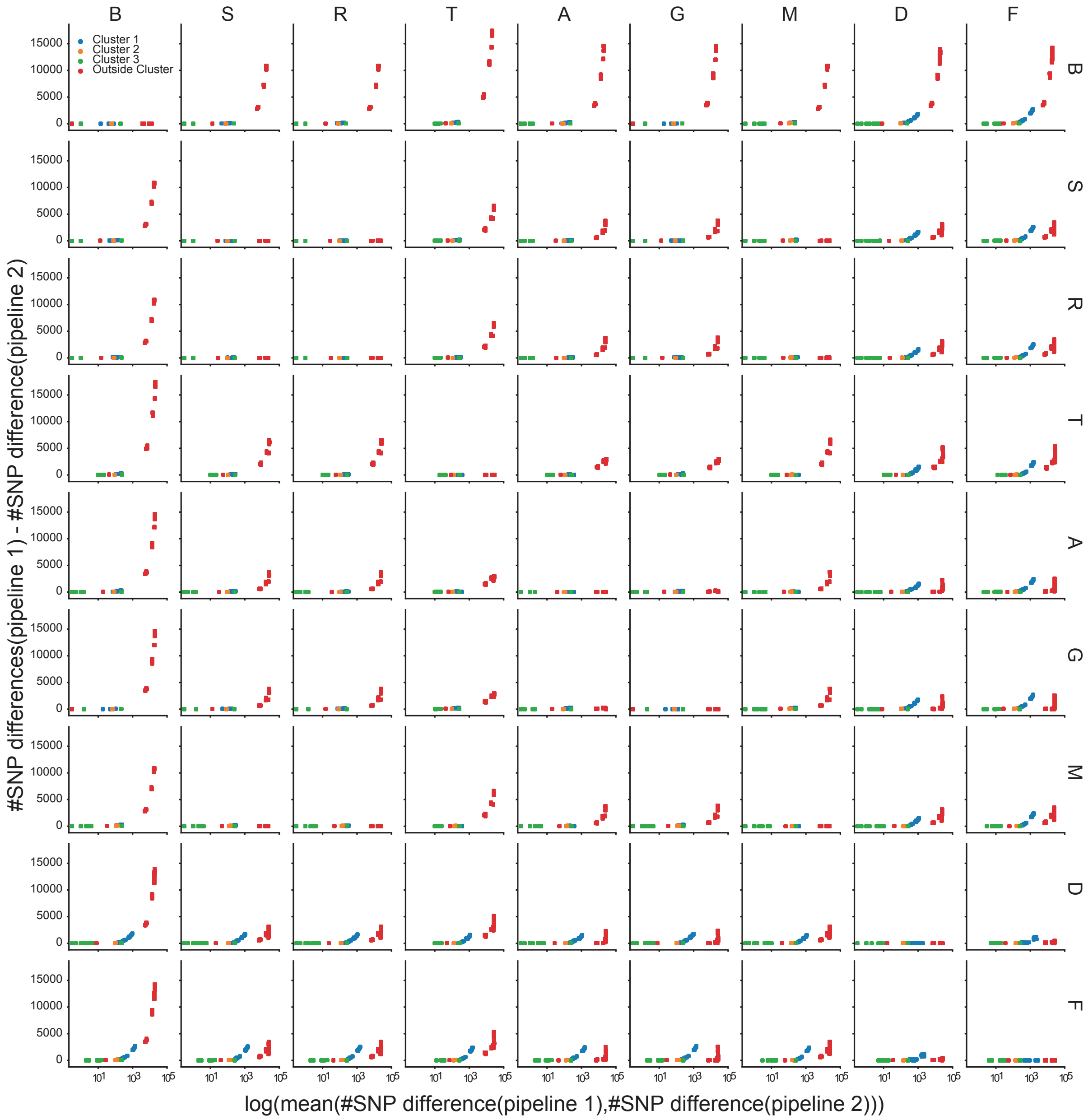

Supplementary Figure 2. *Comparison of pairwise SNP differences across pipelines (increment 2; note that only 9 pipelines from increment 2 reported SNPs (Table 1))*. Every subplot is the comparison between two pipelines, where we plot on the y-axis the number of SNP differences for a pair of strains in the first pipeline, minus the number of SNP differences for that same pair of strains for the second pipeline. Values close to zero indicate that the two pipelines agreed on a similar number of pairwise SNP differences for that pair of strains (see also Fig. 3B, which is essentially another representation of the same data). Data points are represented against the average of the number of SNP differences in the first and second pipeline for the corresponding pair of strains (x-axis).

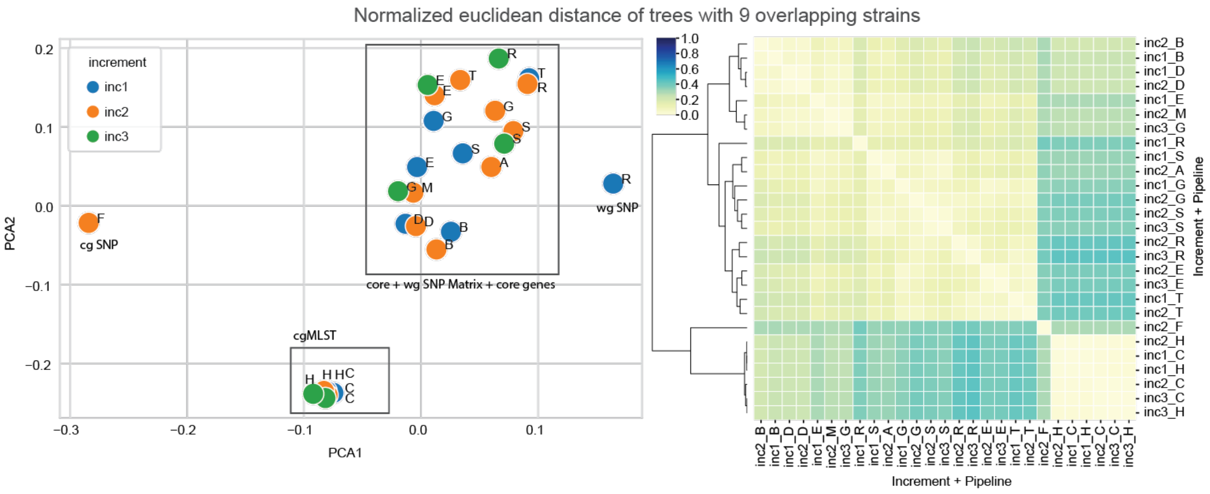

Supplementary Figure 3. (right) Matrix of Euclidean distance between pairs of trees containing only the overlapping nine strains. (left) First two principal components of the distance matrix. We see two distinct clusters that can be separated by the input data used for tree inference.

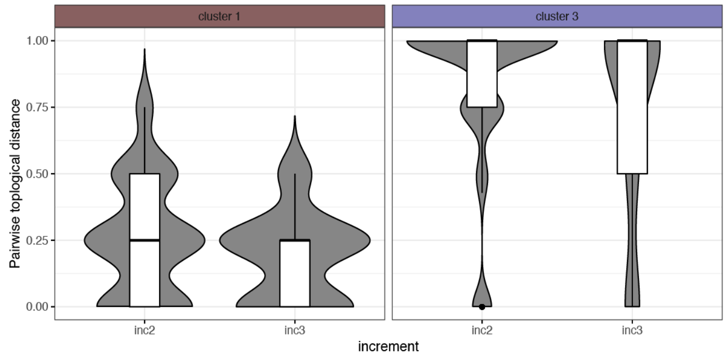

Supplementary Figure 4. Pairwise topological distance of clusters 1 and 3 as defined in **Fig. 4 A**. We see a high dispersion of distances for both clusters.

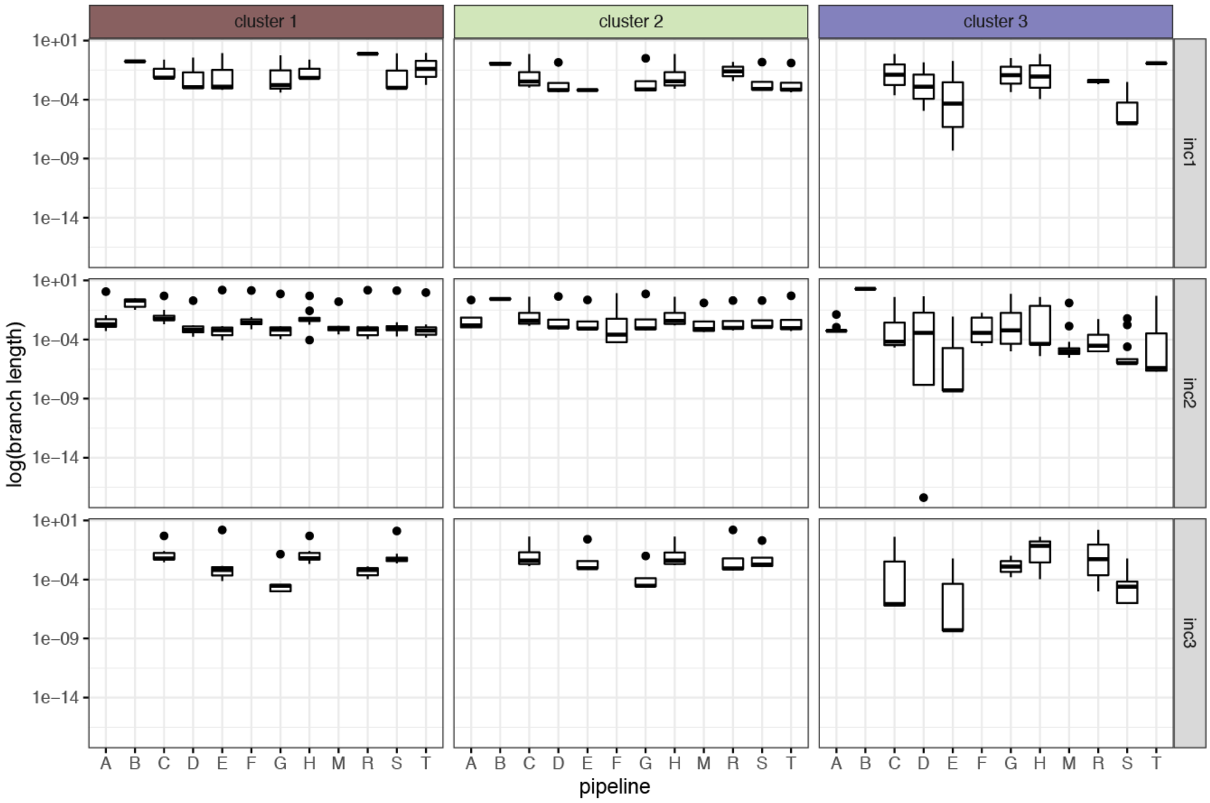

Supplementary Figure 5. Comparison of branch lengths within clusters for all increments using all strains.

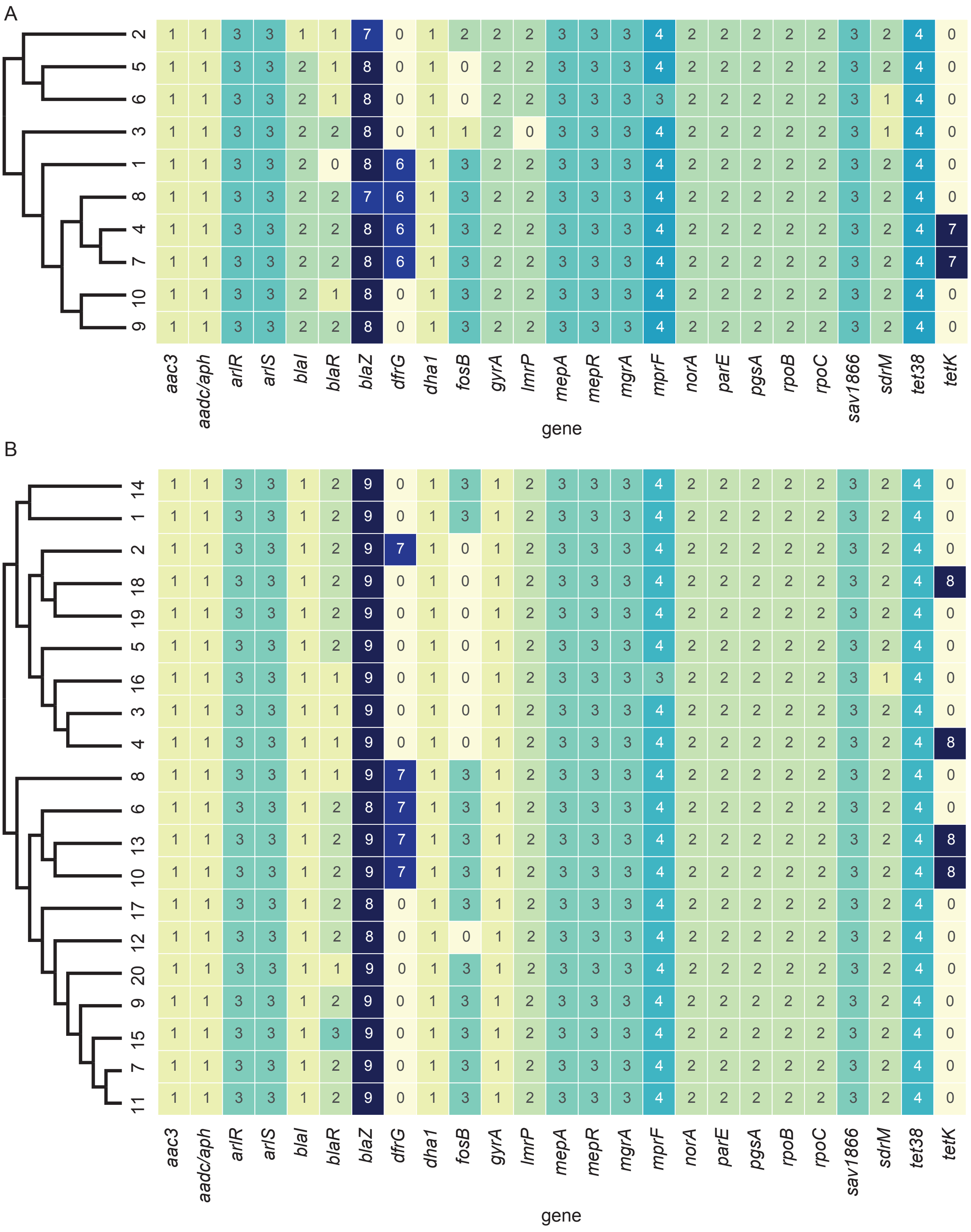

Supplementary Figure 6. Number of pipelines that detect a particular resistance gene for a particular strain. (A) Increment 1 and (B) Increment 2.

Supplementary Tables

Supplementary Table 1: MLST calls in increment 2.

		2		5			8		1		4		10	7	2		6				9	Inc 1 label
**Pipeline, tool**		**1**	**2**	**3**	**4**	**5**	**6**	**7**	**8**	**9**	**10**	**11**	**12**	**13**	**14**	**15**	**16**	**17**	**18**	**19**	**20**	Inc 2 label
B	Bionumerics v. 7.6.3894	U	ST1633	ST152	ST152	ST152	ST15	U	ST5	U	ST15	U	U	ST15	U	U	ST152	ST8	ST152	ST152	U	
C	Seqsphere+	U	ST1633	ST152	ST152	ST152	ST15	U	ST5	U	ST15	U	U	ST15	U	U	ST152	ST8	ST152	ST152	U	
D	CLC Genomics	ST121	ST1633	ST152	ST152	ST152	ST15	U	ST5	U	ST15	U	U	ST15	ST121	U	ST152	S88	ST152	ST152	U	
G	ARIBA 2.11.2	ST121	ST1633	ST152	ST152	ST152	ST15	U	ST5	U	ST15	U	U	ST15	U	U	ST152	ST8	ST152	ST152	U	
M	mlst server 2.10	U	ST1633	U	ST152	ST152	U	U	ST5	U	ST15	U	U	ST15	U	U	ST152	ST8	ST152	ST152	U	
R	mlst server 1.8	ST121	ST1633	ST152	ST152	ST152	ST15	U	ST5	U	ST15	U	U	ST15	ST121	U	ST152	ST8	ST152	ST152	U	
S	mlst server 2.9	U	ST1633	ST152	ST152	ST152	ST15	U	ST5	U	ST15	U	U	ST15	U	U	ST152	ST8	ST152	ST152	U	
T	Seqsphere+	U	ST1633	ST152	ST152	ST152	ST15	U	ST5	U	ST15	U	U	ST15	U	U	ST152	ST8	ST152	ST152	U	

Supplementary Table 2: Phenotypic antibiotic susceptibility tests on the 10 strains from increment 1 (performed by pipeline S). *: identical AST results were found by pipeline G for the overlapping tested antibiotics, except for strain 7 where laboratory G identified tetracycline resistance.

	1	2	3	4	5	6	7	8	9	10
PENICILLINE	R	R	R	R	R	R	R	R	R	R
OXACILLINE	S	S	S	S	S	S	S	S	S	S
GENTAMICINE	S	S	S	S	S	S	S	S	S	S
TOBRAMYCINE	S	S	S	S	S	S	S	S	S	S
TETRACYCLINE	S	S	S	R	S	S	S/R*	S	S	S
TIGECYCLINE	S	S	S	S	S	S	S	S	S	S
ERYTHROMYCIN	S	S	S	S	S	S	S	S	S	S
CLINDAMYCIN	S	S	S	S	S	S	S	S	S	S
NITROFURANTOIN	S	S	S	S	S	S	S	S	S	S
CIPROFLOXACIN	S	S	S	S	S	S	S	S	S	S
LEVOFLOXACIN	S	S	S	S	S	S	S	S	S	S
MOXIFLOXACIN	S	S	S	S	S	S	S	S	S	S
RIFAMPICIN	S	S	S	S	S	S	S	S	S	S
VANCOMYCIN	S	S	S	S	S	S	S	S	S	S
TEICOPLANIN	S	S	S	S	S	S	S	S	S	S
FUSIDIC ACID	S	S	S	S	S	S	S	S	S	S
FOSFOMYCIN	S	S	S	S	S	S	S	S	S	S
MUPIROCIN	S	S	S	S	S	S	S	S	S	S
LINEZOLID	S	S	S	S	S	S	S	S	S	S
MLSB inducible	Negative	Negative	Negative	Negative	Negative	Negative	Negative	Negative	Negative	Negative

Supplementary Materials

Supplementary Material 1: FASTQ datasets for increment 2, including associated pseudonymised epidemiological information. https://doi.org/10.5281/zenodo.3924094

Supplementary Material 2: Assemblies and SNP calls for increment 3, including associated pseudonymised epidemiological information. https://doi.org/10.5281/zenodo.3924110

Supplementary Material 3: Online questionnaire for increment 1. https://doi.org/10.5281/zenodo.3924137

Supplementary Material 4: Analysis scripts and data. https://doi.org/10.5281/zenodo.3924123
